# Evaluation of E-max Porcelain Veneer Failures: A Retrospective Study

**DOI:** 10.7759/cureus.58957

**Published:** 2024-04-24

**Authors:** Rayan Imad, Saba Tahir, Hafsa Alidrissi, Shallen Varma, Lovely M Annamma, Mawada Abdelmagied, Ban A Almudarris, Huda Abutayyem, Mohammad K Alam

**Affiliations:** 1 Dentistry, Irish Wellness and Medical Center, Abu Dhabi, ARE; 2 Dentistry, Spectrum Medical Center, Abu Dhabi, ARE; 3 Prosthodontics, Dr. Rami Hamed Center Dubai Health City, Dubai , ARE; 4 Implantology, Fakeeh University Hospital, Dubai, ARE; 5 Department of Clinical Sciences, College of Dentistry, Ajman University, Ajman, ARE; 6 Clinical Sciences, Center of Medical and Bio-Allied Health Sciences Research, Ajman, ARE; 7 Restorative and Prosthodontic Unit, College of Dentistry, City University, Ajman, ARE; 8 Department of Preventive Dentistry, College of Dentistry, Jouf University, Sakaka, SAU

**Keywords:** chipping, debonding, periodontal health, gingival health, e max veneers

## Abstract

Aim

This retrospective study aimed to evaluate if E-max veneers over five years caused changes in gingival, periodontal health, and veneer failures.

Background

As aesthetic dentistry progresses, dental veneers are becoming increasingly popular in both general and specialized dental practices. Due to technological advancements in dental ceramics and adhesive systems, porcelain veneers have become a highly sought-after solution for improving aesthetics in dental patients. The success of porcelain laminate veneers, a commonly used method for aesthetic restoration, relies on various factors. E-max veneers are frequently utilized, with their long-term durability contingent upon factors such as color stability, resistance to abrasion, as well as good compressive, tensile, and shear strength, along with maintaining marginal integrity.

Methodology

In this study, data was collected through a checklist form used to record clinical parameters. The clinical parameters evaluated were inflammation and bleeding on probing (BOP). The gingival health was evaluated by gingival index, gingival color, texture, and bleeding on probing, and periodontal health was evaluated by the pocket depth and radiographic evaluations. Finally, the veneer was visually inspected for chipping, staining, and debonding history. The score for most of the cases ranged between 0-1, with only 10 cases displaying moderate gingival inflammation and BOP (Gingival Index 2). Siemens Orthopantomogram (OPG) systems were used for radiological evaluation and documentation of cases. E-max porcelain veneers were only included in the research.

Results

Out of 28 patients, each with 6-to-10-unit veneer cases was examined, 18 patients (64.3%) displayed healthy gingival status with no bleeding area recorded in none of the veneers amongst the 6 to 10 units. In 10 patients (35.7%) most of the veneers had inflamed gingival tissue that was bleeding on probing. The majority revealed the presence of stippling (92.9%), absence of recession (96.4%), and pocket depth (67.9%). Half of our participants had their veneer for more than five years (50%) and the majority presented with no significant changes in veneer recorded like marginal staining, debonding, or chipping (89.3%).

Conclusion

Multiple factors such as patient selection, proper treatment planning, and design, including material selection, play a significant role in the long-lasting success of ceramic veneers. The retrospective study indicated that proper oral hygiene measures are vital for the long-term sustainability of E max veneers.

## Introduction

As the field of aesthetics dentistry evolves, dental veneers are increasingly becoming very popular in general and advanced dental practices. With the advancement in the technology of dental ceramics and adhesive systems, porcelain veneers have become an often sought-after treatment for aesthetics. Restorative dentistry must be conservative yet satisfy the patient's aesthetic, biological, and mechanical requirements. Patients are more inclined to choose dental veneers to treat concerns like discoloration, chipping, asymmetry, and diastemas, as it is a conservative restoration. Today, dentists can choose from various materials for dental veneers to match individual clinical cases. While composite veneers require less invasive tooth preparation, porcelain, and ceramic veneers offer better aesthetics and durability while requiring more tooth preparation [1].

The dental veneers are classified based on techniques such as a direct technique with composite resins and an indirect technique with porcelain veneers. The resin composite veneers are the most conservative forms that can mask discolored teeth and modify tooth shape, size, and position [2]. Although these types of restorations are long-lasting, resin composites are still prone to change color and can wear easily compared to ceramic. Composite veneers are more likely to fracture reducing the aesthetic life of the restorations [2]. Over the last decade, porcelain veneers have been introduced as a means of achieving more durable aesthetics. As of now, leucite-reinforced ceramics and lithium disilicate ceramics are commonly recommended for aesthetic veneers due to their optical properties, ability to be acid etched, and being less porous than other ceramics [3]. Due to the low refractory index of these materials, they have an imitable translucent incisal edge enhancing the overall aesthetic result [4]. 

However, with the increasing use of porcelain as a material option for veneers, there is also a concern about how porcelain aﬀects periodontal tissues and general gingival health. A wide consensus exists that porcelain is the most aesthetic and biocompatible material in dentistry yet gingival inflammation is reported [5]. Periodontitis is closely influenced by the periodontal flora growth within the gingival crevicular fluid [6,7]. Several studies concluded that good oral hygiene measures increase the longevity of porcelain veneers with no to minimal eﬀect on periodontal health. The type of finish line of porcelain veneers also affects periodontal health [8]. 

Pippin et al. observed that the location of the restoration concerning the gingival margin also plays a significant part in the gingival response. Compared to metal-ceramic crowns, subgingival marginal preparations of veneers have been shown to have a reduced gingival reaction at the same location [7]. Another factor that can cause gingival inflammation due to plaque accumulation is the increased roughness of glazed porcelain. In another study, plaque retention at the cervical margins of five-year-old porcelain veneers increased slightly due to increased roughness [6]. In our study, the most used veneer material in our department, E-max veneer, was evaluated over five years with a follow-up of routine oral hygiene measures. This study aimed to evaluate if E-max over five years caused changes in gingival, periodontal health, and veneer failures.

## Materials and methods

The study was approved by the Ajman University Ethical Research Committee (Reference number: D-F-H-14-sep). In inclusion criteria, 28 cases with multiple E-max veneers of 6 to 10 units with five-year follow-ups were considered. Only preparation designs were included in the study. Exclusion criteria used were patients with single veneer per arch, veneers damaged by trauma, other dental procedures, patients who underwent any gingival surgical procedures, or recent oral prophylaxis. Data was collected through a checklist form to record clinical parameters for evaluation.

Both Miller's gingival recession classification and Loe and Silness' gingival index were used in evaluating the gingival condition. It was found that the marginal tissue recession in most of the cases did not extend beyond the mucogingival junction with no interproximal tissue loss as measured using the markings on Hu Friedy No.6 periodontal probe (Hu Friedy, Chicago, USA), therefore classified at Grade 1. To determine the gingival index, additional measurements were made media-labially, labially, and disto-labially as described by Loe and Silness' most cases scored 0-1 with only a few showing moderate inflammation and bleeding on probing. Siemens Orthopantomogram (OPG; Siemens, Riyadh, Saudi Arabia) systems were used for radiological evaluation and documentation of cases. According to the three steps of staging and grading periodontal disease published by the American Academy of Periodontology (2018), in veneered cases with pockets exceeding 3 mm, radiographic bone loss was estimated to be no more than 2-3 mm. By subtracting current bone levels from healthy ones, bone loss levels were calculated. The sample size (n) was calculated via the online power and sample size calculator (https://powerandsamplesize.com/Calculators/). The online calculator was used to estimate the minimum per-group sample size, given that the probability level is 0.05, the null hypothesis based on similar studies is 0.5, and the statistical power level of 0.95.

The selected participant's medical and dental histories were reevaluated. The sampling method used in this research was convenience sampling from patients attending the dental clinic with a previous veneer of five years. The consent form was obtained in English and Arabic for Arabic-speaking patients. The information to patients included a detailed explanation of clinical evaluation, data to be collected, how it will be stored, and who will have access to data. The clinical evaluation of the patients was video-demonstrated before proceeding with the clinical examination. The clinical examination included periodontal pocket measurement, gingival recession measurements when applicable, gingival color evaluation, veneer marginal staining, debonding, and chipping. In case the pocket depth exceeded 3 mm a radiographic evaluation was performed. The calibration accuracy of a person’s clinical examination judgment to the actual clinical scenario is counter-checked and verified by another examiner.

Statistical analysis

Compiled data were tabulated using Microsoft Office Word and Excel 2019. Statistical analysis for all descriptive statistics was performed using Statistical Package For The Social Sciences (SPSS) software, version 28 (IBM Corp., Armonk, NY). Spearman’s rho correlation was used to show if there is a strong positive correction between the gingival changes and bleeding on probing (r=0.689) with a significant diﬀerence (p<0.001). The significance level for all tests was p<0.001. The main variables and accordingly the data are demonstrated as frequencies and percentages; furthermore, one sample Chi-square test will be used to check if there is any significant difference from the hypothesized population distribution. P value is set at below 0.05 with a 95% confidence level.

## Results

Out of 28 veneer cases examined in our department, 18 (64.3%) displayed healthy gingival status with no bleeding area recorded and 10 (35.7%) had inflamed gingival tissue with bleeding on probing in some teeth. The majority revealed the presence of stippling (92.9%), the absence of recession (96.4%), and pocket depth (67.9%) (Table 1).

**Table 1:** Frequency table The frequency table indicates all the clinically evaluated factors and the frequency and percentage of the factors present indicate problems associated with the E-max veneers.

Variables	Gingival status	Frequency	Percentage
Clinical Examination Findings: Color	Healthy	18	64.3
	Inflamed	10	35.7
Clinical Examination Findings: Texture	Stippling Present	26	92.9
	Stippling Absent	2	7.1
Clinical Examination Findings: Bleeding	no significant bleeding areas recorded	18	64.3
	bleeding on probing in teeth	10	35.7
Clinical Examination Findings: Recession	Present	1	3.6
	Absent	27	96.4
Clinical Examination Findings: Pocket Depth	Present	9	32.1
	Absent	19	67.9
Date of cementation	within 2 years	6	21.4
	3-5 years ago,	8	28.6
	> 5 years ago,	14	50.0
Post Follow up Veneer status (i.e.: marginal staining, debonded, chipping)	Staining	2	7.1
	Debonding	1	3.6
	no significant changes in veneer recorded	25	89.3

**Figure 1:**
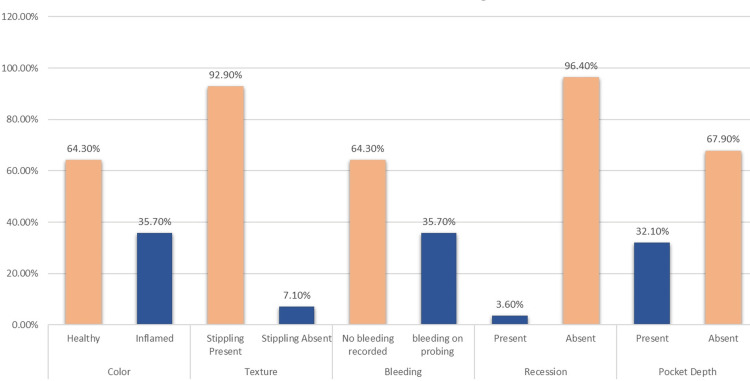
Clinical findings of E-max veneers after five years

These results indicate that minor complications are present with porcelain veneers especially if oral hygiene instructions are not followed apart from other biological factors. All the clinical findings in our study are depicted in Graph (Figure 1).

Half of our participants had their veneer for more than five years (50%) and the majority presented with no significant changes in veneer recorded like marginal staining, debonding, or chipping (89.3%). In one case in our study multiple debonding and staining. (Figures 2a, 2b). Spearman’s rho correlation showed that there is a strong positive correction between the gingival changes and bleeding on probing (r=0.689) with a significant difference (p<0.001) (Table 2).

**Table 2:** Nonparametric correlations sig: Significant

Variables			Colour	Texture	Bleeding	Recession	Pocket Depth	Date of cementation	Veneer status
Spearman's rho	Colour	Correlation Coefficient	1.000	.372	.689	-.258	-.285	.151	-.241
		Sig. (2-tailed)	.	.051	< .001>	.185	.142	.444	.217
	Texture	Correlation Coefficient	.372	1.000	-.207	-.694	-.106	.056	-.831
		Sig. (2-tailed)	.051	.	.291	< .001>	.591	.777	< .001>
	Bleeding	Correlation Coefficient	.689	-.207	1.000	.143	-.125	-.030	.258
		Sig. (2-tailed)	< .001>	.291	.	.466	.525	.879	.185
	Recession	Correlation Coefficient	-.258	-.694	.143	1.000	.280	.104	.577
		Sig. (2-tailed)	.185	< .001>	.466	.	.150	.599	.001
	Pocket Depth	Correlation Coefficient	-.285	-.106	-.125	.280	1.000	-.536	.018
		Sig. (2-tailed)	.142	.591	.525	.150	.	.003	.929
	Date of cementation	Correlation Coefficient	.151	.056	-.030	.104	-.536^**^	1.000	-.149
		Sig. (2-tailed)	.444	.777	.879	.599	.003	.	.448
	Post-follow-up Veneer status	Correlation Coefficient	-.241	-.831	.258	.577	.018	-.149	1.000
		Sig. (2-tailed)	.217	< .001>	.185	.001	.929	.448	.
		N	28	28	28	28	28	28	28

One-Sample Binomial Test showed a significant difference between different patients (p<0.001) concerning gingival texture, recession, and follow-up. Veneer status (i.e., marginal staining, debonded, chipping) in which most of them had healthy and good appearance (Table 3).

**Table 3:** Hypothesis test summary

Serial number	Null Hypothesis	Test	Significance	Decision
1	The categories defined by Clinical Examination Findings: Colour = Inflamed and Healthy occur with probabilities .500 and .500.	One-Sample Binomial Test	.186	Retain the null hypothesis.
2	The categories defined by Clinical Examination Findings: Texture = Stippling Absent and Stippling Present occur with probabilities .500 and .500.	One-Sample Binomial Test	< .001	Reject the null hypothesis.
3	The categories defined by Clinical Examination Findings: Bleeding = no significant bleeding areas recorded and bleeding on probing in some teeth occur with probabilities .500 and .500.	One-Sample Binomial Test	.186	Retain the null hypothesis.
4	The categories defined by Clinical Examination Findings: Recession = Absent and Present occur with probabilities .500 and .500.	One-Sample Binomial Test	< .001	Reject the null hypothesis.
5	The categories defined by Clinical Examination Findings: Pocket Depth = Absent and Present occur with probabilities .500 and .500.	One-Sample Binomial Test	.089	Retain the null hypothesis.
6	The categories of Date of cementation occur with equal probabilities.	One-Sample Chi-Square Test	.156	Retain the null hypothesis.
7	The categories of Post Follow-up Veneer status (i.e.: marginal staining, debonded, chipping) occur with equal probabilities.	One-Sample Chi-Square Test	< .001	Reject the null hypothesis.

Moreover, the current study revealed a significant association between the date of cementation and pocket depth (p=0.016) in which 57.1% of increased pocket depth was reported in patients who had veneer for more than 5 years (Table 4).

**Table 4:** Association between the date of cementation and clinical examination findings of pocket depth

Variables			Pocket Depth		Total	P value
			Present	Absent		
Date of cementation	within two years	Count	0	6	6	0.016
		% within Date	0.0%	100.0%	100.0%	
		% of Total	0.0%	21.4%	21.4%	
	three-five years ago,	Count	1	7	8	
		% within Date	12.5%	87.5%	100.0%	
		% of Total	3.6%	25.0%	28.6%	
	> five years ago	Count	8	6	14	
		% within Date	57.1%	42.9%	100.0%	
		% of Total	28.6%	21.4%	50.0%	
Total		Count	9	19	28	
		% within Date	32.1%	67.9%	100.0%	
		% of Total	32.1%	67.9%	100.0%	

## Discussion

Porcelain laminate veneer research has various articles published on the technical and aesthetic aspects of failure. A dearth of literature still exists on the long-term effects of different types of veneers and their effects on gingival and periodontal status. In the pursuit of restorative perfection, many veneered teeth tend to fail mostly due to preparations encroaching into the biological width space, and to errors in tooth preparations itself. Clinical evidence suggests that the gingival response to porcelain veneers can be excellent, provided all factors, from patient selection to post-operative instructions, are meticulously followed. Because of increased motivation or improved oral hygiene measures, gingival tissues are symptom-free and healthier than before the placement of veneers. Porcelain appears to retain plaque but has been shown to dislodge more quickly than other restorative material surfaces [9]. The four aspects of design that are directly linked to periodontal health are the level of margin placement, margin adaptation, restoration contour, and occlusal factors [10,11]. Evidence-based retrospective studies, ranging from five to 20 years, have documented the success of porcelain veneer therapy and its favorable clinical performance [12,13]. Reports on minor complications, such as the incidence of postoperative sensitivity (greater than 20%) and the incidence of postoperative pulpitis (less than 2.1%) reported by Zhang et al. [14], indicate the caution to be exercised during porcelain veneer therapy. Apart from different fabrication techniques, porcelain veneers can be done on either prepared or unprepared teeth. According to current research, there is no significant difference in the adverse reaction of periodontal tissues to veneers done on prepared and unprepared teeth [14]. The other complications, such as caries, debonding, and ceramic veneer fracture were observed in long-term retrospective studies [12,15-17]. This is consistent with the current study's findings. In the current study, we were able to view twenty-eight cases that shed light on the alteration of periodontal status, particularly, after veneer placement. Several other research papers argue that it is merely the positioning of the restorative margin that makes all the difference [14]. Three levels of restorative margins are well known: Supragingival, Equi-gingival, and Subgingival - Ideally wherever possible, supra gingival followed by Equi-gingival margins are preferable due to its favorable outcomes and minimal damage to the biological width, but studies with no finish lines are also reported [18]. Leevailoj et al. observed that the survival rate of veneer restorations was 97.5%. The remaining 2.5% failure was due to veneer fracture and debonding. They observed higher failure rates in premolar veneers [19].

Contrary views on the effects of prepared and unprepared teeth on gingival and periodontal tissues have been reported. Shaini et al. reported that unprepared porcelain veneers, compared to prepared tooth veneers, had a higher success rate of restoration [20]. Tsubota et al. reported that unprepared porcelain veneers are more likely to be oversized and unaesthetic. They also reported that oversized veneers can lead to plaque and bacterial accumulation causing periodontal disease in the long term [21]. Hence, a larger sample size is required to determine the effect of non-preparation techniques versus preparation techniques. The preparation design studies veneer placement was considered successful in long-term evaluation [22]. The other factors in the success of longevity depend on proper preparation guidelines such as margin placement, maintenance of anatomical contours, harmonious occlusions, and adequate thickness of attached gingiva and biological width within normal limits [23]. Brian LeSage published a step-by-step guide to ceramic veneer preparation techniques with parameters of thickness and space for veneers [24]. Oversized restoration is mostly the effect of insufficient tooth preparation. The technician has no option but to oversize the restoration to maintain a minimum acceptable thickness of the porcelain veneer [25]. Evidence from research indicates that over-contouring causes gingival inflammation [25]. The reason for over-contoured crowns causing gingival inflammation is the cause of plaque buildup and difficulty in cleaning those areas [9]. The commonest reasons for failure amongst the ceramic veneer were cited to be fracture and a higher rate of debonding especially in bruxers [26]. This finding was similar to one of our multiple debonded cases where the patient was a bruxer and smoker, which explains the debonded veneer with extensive staining due to smoking. When a comparison of CAD-CAM (computer-aided-design and computer-aided-manufacturing) fabricated veneers with conventional veneers was reviewed by Badami et al. they observed that both functioned equally well in terms of marginal adaptation [23]. In our study, the majority of cases that showed gingival inflammation were patients who did not follow proper oral hygiene measures or were smokers. The one debonded case was a patient who was a bruxer. All 28 cases had Equi gingival margin preparation.

## Conclusions

As valuable as they are, aesthetic veneer procedures contribute greatly to overall patient smile satisfaction and improved quality of life, especially in the psychological and social aspects. The advancement of micro-minimally invasive technology and advanced material technology has given porcelain veneer treatment a broader scope and more options. Multiple factors such as patient selection, proper treatment planning, and design, including material selection, play a significant role in the long-lasting success of ceramic veneers.

## References

[1] McLaren EA, Whiteman YY (2010). Ceramics: rationale for material selection. Compend Contin Educ Dent.

[2] Peumans M, Van Meerbeek B, Lambrechts P, Vanherle G (1997). The 5-year clinical performance of direct composite additions to correct tooth form and position. I. Esthetic qualities. Clin Oral Investig.

[3] Radz GM (2011). Minimum thickness anterior porcelain restorations. Dent Clin North Am.

[4] Griggs JA (2007). Recent advances in materials for all-ceramic restorations. Dent Clin North Am.

[5] Alrahlah A, Altwaim M, Alshuwaier A (2021). Influence of ceramic lumineers on inflammatory periodontal parameters and gingival crevicular fluid IL-6 and TNF-α levels—A clinical trial. Appl Sci.

[6] Quirynen M, Bollen CM (1995). The influence of surface roughness and surface-free energy on supra- and subgingival plaque formation in man. A review of the literature. J Clin Periodontol.

[7] Pippin DJ, Mixson JM, Soldan-Els AP (1995). Clinical evaluation of restored maxillary incisors: veneers vs. PFM crowns. J Am Dent Assoc.

[8] Alqahtani FI, Algohar A, Alhazzaa A, Dimashkieh A (2019). Gingival health in patients treated with full veneer crown restorations in Al-Riyadh Province, Kingdom of Saudi Arabia. World J Dent.

[9] Kourkouta S, Walsh TT, Davis LG (1994). The effect of porcelain laminate veneers on gingival health and bacterial plaque characteristics. J Clin Periodontol.

[10] Kois JC (1996). The restorative-periodontal interface: biological parameters. Periodontol 2000.

[11] Padbury A Jr, Eber R, Wang HL (2003). Interactions between the gingiva and the margin of restorations. J Clin Periodontol.

[12] Fradeani M, Redemagni M, Corrado M (2005). Porcelain laminate veneers: 6- to 12-year clinical evaluation--a retrospective study. Int J Periodontics Restorative Dent.

[13] Aqlan S, Kheiralla L, El-Naggar G (2023). Clinical performance of ceramic laminate veneers made with Celtra Press and IPS E. Max Press ceramic (randomized controlled clinical trial). J Popul Ther Clin Pharmacol.

[14] Zhang R, Shen L, Xu D, Li X (2021). Analysis of the effects of prepared porcelain veneers and unprepared porcelain veneers on gingival crevicular flora based on high-throughput sequencing. Exp Ther Med.

[15] Chai J, McGivney GP, Munoz CA, Rubenstein JE (1997). A multicenter longitudinal clinical trial of a new system for restorations. J Prosthet Dent.

[16] Gemalmaz D, Ergin S (2002). Clinical evaluation of all-ceramic crowns. J Prosthet Dent.

[17] AlJazairy YH (2021). Survival rates for porcelain laminate veneers: a systematic review. Eur J Dent.

[18] Peris H, Godoy L, Cogolludo PG, Ferreiroa A (2019). Ceramic veneers on central incisors without finish line using bopt in a case with gingival asymmetry. J Clin Exp Dent.

[19] Leevailoj C (2018). The longevity of ceramic veneers: Clinical evaluation of mechanical, biologic and aesthetic performances of ceramic veneers, a 7-year retrospective study. J Dent Assoc Thai.

[20] Shaini FJ, Shortall Ac, Marquis Pm (1997). Clinical performance of porcelain laminate veneers. A retrospective evaluation over a period of 6.5 years. J Oral Rehabil.

[21] Tsubota K (2017). Ten-year clinical observation of a porcelain laminate veneer seated with biological tissue adaptation (BTA) technique. J Oral Sci.

[22] Borges B, Costa GDFD, Assunção IVD (2016). Clinical performance of porcelain laminate veneers with minimal preparation: a systematic review. Int J Exp Dent Sci.

[23] Badami V, Satya Priya M, Vijay L, Kethineni H, Akarapu S, Agarwal S (2022). Marginal adaptation of veneers: a systematic review. Cureus.

[24] Lesage B (2010). Revisiting the design of minimal and no-preparation veneers: a step-by-step technique. J Calif Dent Assoc.

[25] Malament KA, Margvelashvili-Malament M, Natto ZS, Thompson V, Rekow D, Att W (2021). Comparison of 16.9-year survival of pressed acid etched e.max lithium disilicate glass-ceramic complete and partial coverage restorations in posterior teeth: Performance and outcomes as a function of tooth position, age, sex, and thickness of ceramic material. J Prosthet Dent.

[26] Granell-Ruíz M, Agustín-Panadero R, Fons-Font A, Román-Rodríguez JL, Solá-Ruíz MF (2014). Influence of bruxism on survival of porcelain laminate veneers. Med Oral Patol Oral Cir Bucal.

